# Does loneliness lurk in temp work? Exploring the associations between temporary employment, loneliness at work and job satisfaction

**DOI:** 10.1371/journal.pone.0250664

**Published:** 2021-05-03

**Authors:** Eline Moens, Stijn Baert, Elsy Verhofstadt, Luc Van Ootegem

**Affiliations:** 1 Department of Economics, Ghent University, Ghent, Belgium; 2 University of Antwerp, Antwerp, Belgium; 3 Université catholique de Louvain, Louvain-la-Neuve, Belgium; 4 IZA, Bonn, Germany; 5 GLO, Maastricht, The Netherlands; 6 IMISCOE, Rotterdam, The Netherlands; Aalborg University, DENMARK

## Abstract

This research contributes to the limited literature concerning the determinants of loneliness at work, as well as to the literature on psychological outcomes associated with temporary work. More specifically, we are adding to the literature by exploring whether there is an association between working temporarily and loneliness at work and whether loneliness at work partly explains the association between working temporarily and job satisfaction. To this end, we analyse—by means of a mediation model—a unique sample of Flemish employees in the private sector. We find that employees with a temporary contract experience more loneliness at work as opposed to employees with a permanent contract. In addition, we discover that loneliness at work mediates the association between working temporarily and job satisfaction.

## Introduction

At a societal level, loneliness is a hot topic. Even Ministers of Loneliness are popping up (e.g. [1]). Loneliness occurs in all walks of life and society is starting to realise that the working population is not an exception (e.g. [2]). Researchers acknowledge that people can experience loneliness, even when being surrounded by co-workers [3].

Despite its societal relevance, researchers who study loneliness in the workplace agree that the topic has received relatively little attention in the scientific literature (e.g. [3, 4]). Particularly concerning the determinants of loneliness at work, researchers have been advised to take a much broader look at determinants of loneliness at work as opposed to only looking at its personal characteristics like extraversion or shyness (e.g. [5]). This plea was made by Sarah Wright—one of the founders of research concerning loneliness at work—about a decade ago. In response to this plea, also social (e.g. [6]), organisational, and job characteristics (e.g. [7]) have been investigated as determinants of loneliness at work. However, several determinants of professional loneliness, including many job characteristics, still need to be investigated.

Temporary work—defined as dependent employment of limited duration [8]—is one of the unexplored determinants of loneliness at work related to job characteristics. Therefore, our first research question reads as follows: ‘Is there an association between working temporarily and loneliness at work?’. This fills a substantial gap in the literature for several reasons. First, the share of temporary employees in the EU is on the rise so that monitoring the psychosocial consequences of this evolution is important [9, 10]. Second, related concepts to loneliness at work (e.g. social isolation and a lack of social support) have been linked to temporary employment in the literature. De Cuyper and colleagues [11] mention feelings of social isolation amongst temporary employees. According to Byoung-Hoo and Frenkel [12], temporary employees may receive little support from permanent co-workers. George and colleagues [13] identify negative relationships with other colleagues when temporary workers are seen as competitors threatening the workplace security. Wilkin and colleagues [14] show sparser social networks for temporary workers in teams compared to their permanent counterparts. On the contrary, Aleksynska [15] proves social environment to be unaffected by contractual status (and therefore it also does not mediate the relationship between temporary employment and job satisfaction) and Ang and Slaughter [16] find that contractors perceive higher levels of organizational support on average than permanent professionals. Although closely related to loneliness at work, the investigated concepts in these articles (i.e. social isolation, support, relationships, networks and social environment) are conceptually distinct from loneliness at work as they refer to the objective characteristics of a social environment while the feeling of loneliness at work is based on an individual’s perception [3]. To illustrate, it is possible that individuals who feel lonely have just as much social contact with others as individuals who do not report feeling lonely [17]. Answering the first research question will therefore clearly contribute to the literature as, to the best of our knowledge, it has not yet been empirically tested whether temporary workers experience more loneliness at work as opposed to permanent workers.

Concerning the association between temporary employment (versus permanent employment) and job satisfaction, research has resulted in inconsistent findings [18, 19]. Some studies show temporary workers have a lower job satisfaction compared to permanent employees (e.g. [15, 20]), while some studies show the opposite (e.g. [21]) or find no significant difference (e.g. [22]). To gain a better insight in this complex association, De Cuyper and colleagues [18] have invited researchers to investigate its mediators. Mediators provide information on how one variable influences another one [23]. Loneliness at work is such a possible mediator: on the one hand there is a potential association between working temporarily and loneliness at work (see above), on the other hand there are indications of a negative association between loneliness at work and job satisfaction (e.g. [24]). Therefore, our second research question is: ‘Does loneliness at work mediate the association between working temporarily and job satisfaction?’. In other words, we explore whether the association between working temporarily and loneliness at work explains a part of the association between working temporarily and job satisfaction.

We conclude this introduction by emphasizing that our aim in this exploratory research is to discover associations rather than causal relations. Our data does not allow causal inference of the associations in our mediation model. We will return to this at the end of this research article when we discuss the limitations of our study. We are however able to get a powerful first indication of the associations in our research questions by taking into account a multitude of important personal characteristics and job characteristics as control variables, as discussed in the next section.

## Data and method

To answer our research questions, we draw on self-reported information that we obtained from a large-scale survey (N = 1358) among employees in the private sector in Flanders—the northern Dutch speaking part of Belgium—between February and October 2019. We used quota-sampling to achieve similarity of the (univariate) frequency distributions in the Flemish population with respect to (i) working part-time versus full-time, (ii) educational level, (iii) gender and (iv) age.

The survey followed the rules of the ethical code at Ghent University in full. The human data in our survey was collected completely based on self-determination and the respondents had the right to stop participating at any moment without the need for justification. The goal and content of the survey were clearly communicated, as was the information that Ghent University was the responsible organizer of the survey. The contact details of the Data Protection Officer of Ghent University are easily accessible on the website of the university. The individuals responsible for the data collection, who acted on behalf of Ghent University, were personally available in case of any further questions or requests of further information on the goal, content and data processing of the survey.

In the questionnaire, the respondents were asked to indicate their type of contract: temporary (code 1) or permanent (code 0). We also asked the respondents to express their job satisfaction from 0 (not satisfied at all) to 10 (totally satisfied). Also, loneliness at work was measured by means of a short version of the R-UCLA loneliness scale [25], which we adapted to the work context. That is, respondents indicated from strongly disagree (1) to strongly agree (5) to which extent they agreed with the following four statements: ‘I feel in tune with the people around me at work’, ‘No one at work really knows me well’, ‘I can find companionship at work when I want it’ and ‘The people at work are not there for me’. Based on the (standardised) answers to these four items a loneliness factor was created (by means of a principal component analysis), which ranges from −1.81 to 4.74 (M = 0.00, SD = 1,00) (Cronbach’s alpha = 0.764).

**Fig 1:**
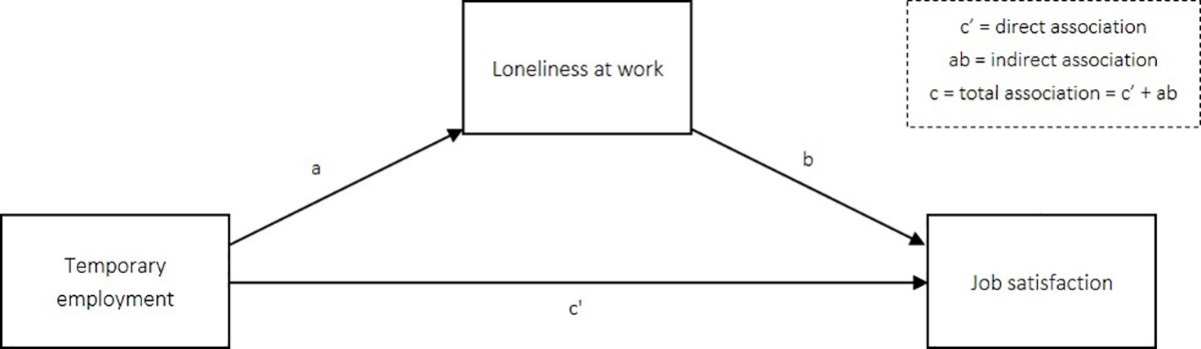
Mediation model.

As depicted in Fig 1, the three variables described above are at the core of our mediation model. We analyse this mediation model following the procedure discussed in Hayes [23]. To find an answer to our first research question, we examine the association between working temporarily and loneliness at work (association *a*). To answer our second research question, we estimate the indirect association between working temporarily and job satisfaction through loneliness at work (association *ab*).

Below, we discuss three versions of this mediation model (A, B and C) that differ only in the number of control variables added to the model. A description of all the control variables gathered in the questionnaire can be found in Table 1. Besides the aforementioned three main variables, we also asked respondents about their gender, age, religion, place of residence (rural/urban), education level and HEXACO personality traits (Honesty-humility (H), emotionality (E), extraversion (X), agreeableness (A), conscientiousness (C) and openness to experience (O)). We add these personal characteristics, which we assume to be predetermined and thereby strictly exogenous, as control variables in model A. Also other socio-demographic information was surveyed in view of this analysis, namely whether respondents are in a relationship, whether they have children and whether they live with someone else (partner, children or others). Together with satisfaction with social life, satisfaction with family life and satisfaction with health, these socio-demographic characteristics are added as extra control variables assumed to be exogenous in model B. In version C of our model, we also added available job characteristics as control variables, which we assume to be endogenous. More concretely, variables capturing whether the respondents work full-time or part-time, whether they have a supervisory position, whether they have interaction outside the organisation (e.g. with customers or suppliers), the job complexity and specialised knowledge required in their job, their job tenure and their amount of days working from home were added as extra control variables in model C.

**Table 1:** Variable specification of the control variables.

Variable	Description
***A*. *Indisputably exogenous control variables***	
Male gender	1 if the employee is male, 0 otherwise.
Age	Age of the employee.
Highest education level	Three dummy variables: lower secondary education, bachelor and master.
Personality traits	Six scale variables: honesty-humility, emotionality, extraversion, agreeableness, conscientiousness, openness to experience (7-point semantic differential scale: opposite personality traits at each end of the scale).
Religious	1 if religious, 0 otherwise.
Place of residence: rural	1 if rural, 0 otherwise.
***B*. *Presumably exogenous control variables***	
Relationship	1 if the employee is in a relationship, 0 otherwise.
Children	1 if the employee has at least one child, 0 otherwise.
Living together	1 if the employee lives together with at least one other person, 0 otherwise.
Good health	Score from 0 (totally do not agree) to 10 (totally agree).
Good social life	Score from 0 (totally do not agree) to 10 (totally agree).
Good family life	Score from 0 (totally do not agree) to 10 (totally agree).
***C*. *Presumably endogenous control variables***	
Full-time or part-time	1 if the employee works full-time, 0 otherwise.
Job tenure	Amount of time in the current job, in the same organisation (in years).
Homeworking	Amount of days working from home.
Job complexity	4 dummy variables: totally do not agree, do not agree, agree, totally agree (reference category = neutral).
Specialised knowledge	4 dummy variables: totally do not agree, do not agree, agree, totally agree (reference category = neutral).
Interaction outside the organisation	4 dummy variables: totally do not agree, do not agree, agree, totally agree (reference category = neutral).
Supervisory position	4 dummy variables: totally do not agree, do not agree, agree, totally agree (reference category = neutral).

## Results

Panel A of Table 2 shows the results of the mediation analysis for model A, i.e. when controlling for the indisputably exogenous variables only. The significant coefficient for *a* gives us a clear answer to our first research question: employees with a temporary contract in our sample experience more loneliness at work. The average loneliness at work score amongst temporary workers is 0.32 units (SE = 0.11) higher than amongst permanent workers.

**Table 2:** Results of the mediation analysis.

	a		c		b		ab		c’	
Model A: Indisputably exogenous control variables	0.32***	(0.11)	−0.36**	(0.16)	−0.42***	(0.04)	−0.14***	(0.05)	−0.22	(0.15)
Model B: Indisputably exogenous control variables + presumably exogenous control variables	0.28***	(0.11)	−0.26*	(0.16)	−0.38***	(0.04)	−0.11***	(0.04)	−0.15	(0.15)
Model C: Indisputably exogenous control variables + presumably exogenous control variables + presumably endogenous control variables	0.23**	(0.11)	−0.13	(0.16)	−0.37***	(0.04)	−0.09**	(0.04)	−0.04	(0.15)

Notes. The presented results are non-standardised estimation coefficients following the PROCESS procedure as described in Hayes [23]. Standard errors are between parentheses. As proposed by Hayes [23], standard errors for *ab* are based on 10.000 bias-corrected bootstrap samples; standard errors for *a*, *c*, *b* and *c’* are based on the normal theory approach.

*** (**) ((*)) indicate significance at the 1% (5%) ((10%)) significance level.

We also see in panel A of Table 2 that employees with a temporary contract experience a lower average job satisfaction (*c* = −0.36, SE = 0.16). To find out whether this association is partly mediated by loneliness at work (our second research question), we first examine association *b*. The significant coefficient for *b* shows that an employee that scores one unit higher on the loneliness at work scale is estimated to be 0.42 units (SE = 0.04) lower in job satisfaction compared to another employee with the same type of contract. Multiplying *a* and *b* leads to a significantly negatively mediated association *ab*: temporary employees score 0.14 units (SE = 0.05) lower on the job satisfaction scale as a result of the association between temporary work and loneliness at work, that in turn is associated with lower job satisfaction. So, the total association between working temporarily and job satisfaction (*c* = −0.36, SE = 0.16), is composed of a (non-significant) direct association (*c’* = −0.22, SE = 0.15) as well as a significant indirect association (*ab* = −0.14, SE = 0.05) via loneliness at work. Consequently, we also find a clear answer to our second research question: loneliness at work indeed partly explains the association between working temporarily and job satisfaction in our research sample.

Adding more control variables to the model lowers the size of the coefficients, but it does not change the answers to our research questions. As can be seen in panel B and C of Table 2, when adding the aforementioned control variables assumed to be exogenous (*a* = 0.28, SE = 0.11) as well as when adding the presumably endogenous job characteristics (*a* = 0.23, SE = 0.11), the conclusion that employees with a temporary contract experience more loneliness at work remains valid. What the indirect association is concerned, the model extended with the most likely exogenous control variables (*ab* = −0.11, SE = 0.04) as well as the most extended model (*ab* = −0.09, SE = 0.04), also confirm that loneliness at work mediates the association between working temporarily and job satisfaction.

As mediation variable in our primary analysis outlined above, we employ the short version of the Revised UCLA Loneliness Scale (R-UCLA). As an extension, i) we investigate whether our results are robust to the exclusion of the items from the loneliness at work scale that might potentially be seen as endogenous ii) we investigate whether a certain subdimension of this scale is more influential. First, as argued by a reviewer of a former version of this article, one could argue that particular items of the loneliness at work factor (‘The people at work are not there for me’ and ‘I feel in tune with the people around me at work’) could be endogenous with respect to temporary employment in a sense that they might be related to the insecurity at work causing an employment strategy with temporary instead of permanent contracts. Therefore, we re-estimate our models excluding these variables (one by one and jointly). S1 Table shows that our results are robust to these alternative specifications of our mediation variable. Second, we investigate whether a certain subdimension of the loneliness at work scale is more influential in a secondary analysis. Russell and colleagues [25] recommend researchers who want a shortened version of the loneliness scale to use the four items (which we adapted to the work context): ‘I feel in tune with the people around me at work’, ‘No one at work really knows me well’, ‘I can find companionship at work when I want it’ and ‘The people at work are not there for me’. In our primary analysis we consider this short version of the Revised UCLA Loneliness Scale (R-UCLA) as a unidimensional measure of loneliness, as intended by Russell and colleagues [25] and later confirmed by Russell [26]. McWhirter [27] however discerns multiple dimensions in the Revised UCLA Loneliness Scale. The items ‘No one at work really knows me well’ and ‘The people at work are not there for me’ belong to the dimension ‘intimate others’, while ‘I feel in tune with the people around me at work’ and ‘I can find companionship at work when I want it’ belong to the dimension of loneliness related to a lack of belonging to the affiliative environment. S2 Table shows that the subdimension ‘intimate others’ is central in our model, while working temporarily and the subdimension ‘affiliative environment’ are not associated. This finding is robust when only considering the item ‘No one at work really knows me well’ (and thus omitting the item ‘The people at work are not there for me’ which could potentially be endogenous).

Finally, we want to elucidate the role of job tenure in our mediation model. This is essential as job tenure and temporary employment are undeniably intertwined. First, we employ alternative specifications of job tenure as control variables in robustness analyses. Claiming an association between temporary employment and loneliness at work is only possible when taking job tenure into account as control variable. We have to rule out that the observed association is due to working for a limited time in the job rather than the contract type. As controlling for job tenure is crucial, we not only control for it linearly in our primary analysis outlined above, we also investigate three alternative specifications of job tenure as control variables in robustness analyses (with ln-transformation of job tenure as control variable, with job tenure and job tenure squared included jointly as control variables and with job tenure as categorical variable (i.e. two dummies of job tenure as control variables)). The results (which can be found in S3 Table) are robust to replacing the linear control job tenure in model C by these three alternative specifications of job tenure. To further elucidate the role of job tenure, we performed the moderated mediation model depicted in S1 Fig as an extension to our primary analysis. S4 Table shows that job tenure does not moderate association c’, nor does it moderate association a. The latter indicates that the association between temporary employment and loneliness at work does not differ in size or strength as a function of job tenure [23]. This strengthens the conclusion that working temporarily and loneliness at work are associated in our sample, regardless of how long a temporary employee works at the company.

## Conclusion

To summarise, this research contributed to the limited literature concerning the determinants of loneliness at work, which is a widespread, topical phenomenon. In addition, we made a contribution to the literature on psychological outcomes associated with temporary work. More concretely, to the best of our knowledge, we were the first to empirically conclude that temporary employees experience more loneliness at work as opposed to permanent employees. Moreover, we shed light on the complex association between working temporarily and job satisfaction by concluding that loneliness at work mediates this association. We were able to do so by analysing—by means of a mediation model—a quota sample of Flemish employees in the private sector. The answers to our research questions were robust to controlling for various sets of relevant control variables.

We end this letter by acknowledging some limitations of this exploratory research and formulating related directions for future research. First, we did not take into account the heterogeneity amongst temporary contracts (e.g. volition, contract duration or the number of parties involved), although it has been mentioned as one of the explanations for the inconsistent research results on temporary employment [18]. Follow-up research should refine our explanatory results by discovering whether the current research results are valid for different types of temporary contracts. Second, our data did not allow causal inference of the associations in our mediation model. Despite the fact that the answers to our research questions stood their ground when taking into account an extended list of control variables, it is still possible that non-observed, confounding variables exist (e.g. company size or sector). To rule this out, exogenous variation in (instruments of) both temporary employment and loneliness at work is needed. Since this will be very hard to accomplish simultaneously, we are in favour of follow-up research that focusses on the investigation of the causal relationships of our mediation model one by one.

## Supporting information

S1 Fig Moderated mediation model. (DOCX)

S1 Table Results of the mediation analysis with exclusions from the loneliness at work factor. (DOCX)

S2 Table Results of the mediation analysis with subdimensions from the loneliness at work factor. (DOCX)

S3 Table Alternative specifications of control variable job tenure in model C. (DOCX)

S4 Table Results of the moderated mediation analysis. (DOCX)
