# Supplementary material for: Does loneliness lurk in temp work? Exploring the associations between temporary employment, loneliness at work and job satisfaction
Source: PLoS One. 2021 May 3;16(5):e0250664. doi: 10.1371/journal.pone.0250664 (PMC8092765; doi:10.1371/journal.pone.0250664)
Supplement: S1 Fig — (DOCX) [file pone.0250664.s001.docx]

**S1 Fig.** Moderated mediation model

c’ = direct association

ab = indirect association

c = total association = c’ + ab

c'

a

Temporary employment

Loneliness at work

Job satisfaction

b

Job tenure
