# Supplementary material for: Does loneliness lurk in temp work? Exploring the associations between temporary employment, loneliness at work and job satisfaction
Source: PLoS One. 2021 May 3;16(5):e0250664. doi: 10.1371/journal.pone.0250664 (PMC8092765; doi:10.1371/journal.pone.0250664)
Supplement: S2 Table — (DOCX) [file pone.0250664.s003.docx]

**S2 Table.** Results of the mediation analysis with subdimensions from the loneliness at work factor

|  |  | **a** | | **c** | | **b** | | **ab** | | **c'** | |
| --- | --- | --- | --- | --- | --- | --- | --- | --- | --- | --- | --- |
| ***Panel A: ‘No one at work really knows me well’ and ‘The people at work are not there for me’ (belonging to subdimension ‘intimate others) as mediator*** | | | | | | | | | | | |
| Model A: Indisputably exogenous control variables |  | 0.42*** | (0.11) | −0.35** | (0.16) | −0.40*** | (0.04) | −0.17*** | (0.05) | −0.18 | (0.16) |
| Model B: Indisputably exogenous control variables + presumably exogenous control variables |  | 0.38*** | (0.11) | −0.26* | (0.16) | −0.35*** | (0.04) | −0.13*** | (0.04) | −0.12 | (0.15) |
| Model C: Indisputably exogenous control variables + presumably exogenous control variables + presumably endogenous control variables |  | 0.33*** | (0.11) | −0.12 | (0.16) | −0.34*** | (0.04) | −0.11*** | (0.04) | −0.01 | (0.15) |
| ***Panel B: ‘I feel in tune with the people around me at work’ and ‘I can find companionship at work when I want it’ (belonging to subdimension ‘affiliative environment) as mediator*** | | | | | | | | | | | |
| Model A: Indisputably exogenous control variables |  | 0.13 | (0.11) | −0.36** | (0.16) | −0.33*** | (0.04) | −0.04 | (0.04) | −0.32** | (0.16) |
| Model B: Indisputably exogenous control variables + presumably exogenous control variables |  | 0.11 | (0.11) | −0.26* | (0.16) | −0.29*** | (0.04) | −0.03 | (0.03) | −0.23 | (0.15) |
| Model C: Indisputably exogenous control variables + presumably exogenous control variables + presumably endogenous control variables |  | 0.06 | (0.11) | −0.13 | (0.16) | −0.28*** | (0.04) | −0.02 | (0.03) | −0.11 | (0.15) |
| ***Panel C: ‘No one at work really knows me well’ (belonging to subdimension ‘intimate others) as mediator*** | | | | | | | | | | | |
| Model A: Indisputably exogenous control variables |  | 0.43*** | (0.10) | −0.36** | (0.16) | −0.34*** | (0.04) | −0.15*** | (0.04) | −0.21 | (0.16) |
| Model B: Indisputably exogenous control variables + presumably exogenous control variables |  | 0.40*** | (0.10) | −0.26* | (0.16) | −0.29*** | (0.04) | −0.12*** | (0.04) | −0.14 | (0.15) |
| Model C: Indisputably exogenous control variables + presumably exogenous control variables + presumably endogenous control variables |  | 0.33*** | (0.11) | −0.13 | (0.16) | −0.27*** | (0.04) | −0.09*** | (0.03) | −0.04 | (0.15) |
| Notes. The presented results are non-standardised estimation coefficients following the PROCESS procedure as described in Hayes [23]. Standard errors are between parentheses. As proposed by Hayes [23], standard errors for *ab* are based on 10.000 bias-corrected bootstrap samples; standard errors for *a*, *c*, *b* and *c’* are based on the normal theory approach. *** (**) ((*)) indicate significance at the 1% (5%) ((10%)) significance level. | | | | | | | | | | | |
