# Supplementary material for: Does loneliness lurk in temp work? Exploring the associations between temporary employment, loneliness at work and job satisfaction
Source: PLoS One. 2021 May 3;16(5):e0250664. doi: 10.1371/journal.pone.0250664 (PMC8092765; doi:10.1371/journal.pone.0250664)
Supplement: S3 Table — (DOCX) [file pone.0250664.s004.docx]

**S3 Table.** Alternative specifications of control variable job tenure in model C

| Model C: Indisputably exogenous control variables + presumably exogenous control variables + presumably endogenous control variables |  | **a** | | **c** | | **b** | | **ab** | | **c'** | |
| --- | --- | --- | --- | --- | --- | --- | --- | --- | --- | --- | --- |
| Job tenure (linear) as control variable (benchmark specification) |  | 0.23** | (0.11) | −0.13 | (0.16) | −0.37*** | (0.04) | −0.09** | (0.04) | −0.04 | (0.15) |
| Ln-transformation of job tenure as control variable |  | 0.20* | (0.11) | −0.13 | (0.16) | −0.37*** | (0.04) | −0.07* | (0.04) | −0.05 | (0.15) |
| Job tenure and job tenure squared included jointly as control variables |  | 0.22** | (0.11) | −0.09 | (0.16) | −0.36*** | (0.04) | −0.08** | (0.04) | −0.01 | (0.15) |
| Two dummies of job tenure as control variables: 1 year or less (reference), between 1 to 2 years and more than 2 years job tenure |  | 0.22** | (0.11) | −0.14 | (0.16) | −0.37*** | (0.04) | −0.08** | (0.04) | −0.06 | (0.15) |
| Notes. The presented results are non-standardised estimation coefficients following the PROCESS procedure as described in Hayes [23]. Standard errors are between parentheses. As proposed by Hayes [23], standard errors for *ab* are based on 10.000 bias-corrected bootstrap samples; standard errors for *a*, *c*, *b* and *c’* are based on the normal theory approach. *** (**) ((*)) indicate significance at the 1% (5%) ((10%)) significance level. | | | | | | | | | | | |
