# Supplementary material for: Does loneliness lurk in temp work? Exploring the associations between temporary employment, loneliness at work and job satisfaction
Source: PLoS One. 2021 May 3;16(5):e0250664. doi: 10.1371/journal.pone.0250664 (PMC8092765; doi:10.1371/journal.pone.0250664)
Supplement: S4 Table — (DOCX) [file pone.0250664.s005.docx]

**S4 Table.** Results of the moderated mediation analysis

|  |  | **a** | | **b** | | **c'** | | **a_M_** | | **c'_M_** | |
| --- | --- | --- | --- | --- | --- | --- | --- | --- | --- | --- | --- |
| Model A: Indisputably exogenous control variables |  | 0.33** | (0.13) | −0.42*** | (0.04) | −0.40** | (0.19) | −0.01 | (0.02) | 0.05 | (0.03) |
| Model B: Indisputably exogenous control variables + presumably exogenous control variables |  | 0.28** | (0.13) | −0.37*** | (0.04) | −0.30 | (0.19) | −0.01 | (0.02) | 0.04 | (0.03) |
| Model C: Indisputably exogenous control variables + presumably exogenous control variables + presumably endogenous control variables |  | 0.26** | (0.13) | −0.37*** | (0.04) | −0.16 | (0.19) | −0.01 | (0.02) | 0.03 | (0.03) |
| Notes. The presented results are non-standardised estimation coefficients following the PROCESS procedure as described in Hayes [23]. *a_M_* and *c’_M_* are the coefficients belonging to the interaction between temporary employment and job tenure respectively in the regression equation of loneliness at work and in the regression equation of job satisfaction. Standard errors are between parentheses. As proposed by Hayes [23], standard errors for *a*, *b* and *c’* are based on the normal theory approach. *** (**) ((*)) indicate significance at the 1% (5%) ((10%)) significance level. | | | | | | | | | | | |
